# Cinobufacini injection suppresses the proliferation of human osteosarcoma cells by inhibiting PIN1-YAP/TAZ signaling pathway

**DOI:** 10.3389/fphar.2023.1081363

**Published:** 2023-03-17

**Authors:** Yuru Chen, Yanyan Wang, Yu Zhai, Ye Yuan, Junhong Wang, Yajing Jin, Lingling Dang, Liming Song, Changbao Chen, Yu Wang

**Affiliations:** ^1^ School of Integrative Medicine, Tianjin University of Traditional Chinese Medicine, Tianjin, China; ^2^ State Key Laboratory of Component-Based Chinese Medicine, Tianjin, China; ^3^ Department of Joint Surgery, Tianjin Hospital, Tianjin University, Tianjin, China; ^4^ Department of Spinal Surgery, Tianjin Hospital, Tianjin University, Tianjin, China

**Keywords:** cinobufacini injection, osteosarcoma, PIN1, YAP, TAZ

## Abstract

Cinobufacini injection (CI), an aqueous extract of *Cutis Bufonis*, is clinically used for cancer therapy in China, but its molecular mechanism for the treatment of osteosarcoma (OS) remains unclear. We constructed U2OS ectopic subcutaneous tumor model to verify the anti-OS effect of CI *in vivo*. Meanwhile, cell proliferation of U2OS and MG63 cells was monitored *in vitro* using the CCK-8 assay, colony formation and morphological changes. Cell cycle arrest and apoptosis were detected by flow cytometry and western blot, which showed that CI significantly inhibited proliferation, induced cell cycle arrest and apoptosis in human OS cells. The further RNA-seq results identified that the Hippo signaling pathway was involved in the anti-OS effect of CI. YAP/TAZ are two major components of the Hippo pathway in breast cancer and are positively regulated by prolyl isomerase PIN1, we assessed their role in OS using both clinicopathological sections and western blots. CI also inhibited PIN1 enzyme activity in a dose-dependent manner, which resulted in impaired PIN1, YAP, and TAZ expression *in vitro* and *in vivo*. Additionally, 15 potential compounds of CI were found to occupy the PIN1 kinase domain and inhibit its activity. In summary, CI plays an anti-OS role by down-regulating the PIN1-YAP/TAZ pathway.

## 1 Introduction

The osteosarcoma (OS) is a highly malignant primary bone tumor. Typically, it occurs in children, adolescents, and young adults, with high aggression, high metastatic potential, poor prognosis, and high short-term mortality (Otoukesh et al., 2018; Saraf et al., 2018). Even when several effective interventions are employed for OS, long-term survival does not improve, especially for patients with multi-drug resistance, recurrences, or lung metastases (Wang et al., 2018). In order to achieve more favorable clinical outcomes and reduce mortality, it is crucial to develop less toxic and more effective alternative therapies.

The dried skin of *Bufo bufo gargarizans Cantor*, Cutis Bufonis, is believed to clear heat, detoxify, induce diuresis, and eliminate edema (LI et al., 2014; Xu et al., 2019). Cinobufacini injection (CI), extracted from Cutis Bufonis, is commonly used in cancer treatment, including OS (Huang et al., 2012; Yang et al., 2015; Ma et al., 2018). However, its molecular mechanisms and the potential bioactive components of OS therapy remain unclear, which limits its clinical application and promotion.

As the first identified tumor suppressor signaling pathway in *Drosophila*, Hippo regulates cell proliferation and apoptosis in mammals, which is crucial to the development of most solid tumors (Avruch et al., 2012; Liu et al., 2018). As downstream effect factors in Hippo pathway, the transcriptional co-activators Yes-associated protein 1 (YAP) and its paralogue the transcriptional co-activator with PDZ-binding motif (TAZ) are involved in cell proliferation, drug resistance and many other tumorigenic processes (Zhang et al., 2015). Notably, Hippo signaling pathway regulates proliferation, apoptosis, invasion, and metastasis of OS cells (Deel et al., 2015; Kovar et al., 2020; Rothzerg et al., 2021; Zhan et al., 2021), and YAP was elevated in OS patients with poor staging (Zhang et al., 2013). Peptidyl-prolyl cis-trans isomerase NIMA-interacting 1 (PIN1) can regulate the activity of Hippo pathway through interaction with Hippo components YAP and TAZ oncoproteins as novel binding partner of PIN1 (Khanal et al., 2019). PIN1 can interact with YAP/TAZ in a phosphorylation-independent manner and WW domain of PIN1 is necessary for this interaction (Khanal et al., 2019). PIN 1 also plays a critical role in the nuclear translocation of TAZ contributes to the dysregulation of Hippo signaling, leading to oncogenic signaling (Khanal et al., 2019; Kim et al., 2021). There is growing evidence that abnormal YAP/TAZ activation is associated with tumors development (Zanconato et al., 2015; Gregorieff and Wrana, 2017). YAP/TAZ overexpression in OS is a potential therapeutic target (Morice et al., 2020), and we have shown that CI inhibits the proliferation of triple negative breast cancer through the PIN1-TAZ signaling pathway (Kong et al., 2022). As a necessary consequence, we’d like to see whether CI could treat OS by inhibiting PIN1-YAP/TAZ pathway.

In this context, the therapeutic effects of CI on OS were investigated by constructing an ectopic subcutaneous tumor model of U2OS *in vivo* and comparing the cell proliferation of U2OS and MG63 cells *in vitro* using the CCK-8 assay, colony formation, and morphological changes. Flow cytometry and western blot confirmed that CI inhibited cell cycle arrest and apoptosis in U2OS and MG63. Furthermore, combined with RNA-seq analysis, we figured out the potential molecular pathway, including the PIN1-YAP/TAZ signaling cascade. Notably, either PIN1 or YAP/TAZ plays an essential role in OS solely, but PIN1-YAP/TAZ pathway has been poorly addressed so far (Zhou et al., 2013; Yang et al., 2014; Deel et al., 2015; Kovar et al., 2020; Rothzerg et al., 2021). In this study, we first highlight the key aspects of high PIN1-YAP/TAZ expression in OS patients, immunohistochemically, which provide a reference for the pathogenesis and clinical treatment of OS. Our study proposed and proved that CI played an anti-OS role by down-regulating the PIN1-YAP/TAZ pathway.

## 2 Manuscript formatting

### 2.1 Materials and methods

#### 2.1.1 Cell culture and reagents

Human OS cell lines MG63, U2OS were obtained from the ATCC (United States). They were cultured at 37°C with DMEM (BI, Israel) supplemented with 10% FBS and 1% penicillin/streptomycin in incubators with humidified air and 5% CO_2_-supplemented air. Anhui Huarun Jinchan Pharmaceutical Co., Ltd. (Anhui, China) supplied CI (Cat. Z34020273, Lot.200,505-2).

#### 2.1.2 Ectopic subcutaneous tumor model

The Animal Care and Use Committee of Tianjin University of Traditional Chinese Medicine approved all animal experiments. U2OS cells (1 × 10^7^ in 100 μLPBS) were subcutaneously injected into four to 6-weeks BALB/c-nude male mice. The mice were divided into four groups at random (model: saline, CI low dose: 0.25 g/kg; CI high dose: 0.5 g/kg and Dox (Doxorubicin): 1 mg/kg) when their average tumor size reached about 100 mm^3^. The longest diameter (a) and shortest diameter (b) of tumors and mouse body weight were measured every 3 days. Calculation of tumor volume: volume (mm^3^) = 1/2 × (a × b^2^).

#### 2.1.3 Assay of cell viability and observation of morphology

An assay for cell viability was performed using the Cell Counting Kit-8 (CCK-8) (Dojindo, Japan). U2OS and MG63 (2 × 10^3^ cells/well) cells were treated with CI (6.25, 12.5, 25, 50, 100, and 500 μg/mL) for 24 and 48 h. Afterwards, cells were washed with PBS two times. Fresh medium (90 µL) and CCK-8 reagent (10 µL) were added and incubated at 37°C for 1 h. The absorbance was determined by Spark microplate reader (Tecan, Männedorf, Switzerland) at 450 nm. For observation of morphology, cells were treated with different concentrations CI for 12, 24, and 48 h, observed under ECLIPSE Ci-L microscope (Nikon, Tokyo, Japan) and photographed.

#### 2.1.4 Colony formation assay

U2OS and MG63 cells were inoculated into 6-well plates and cultured overnight. Following this, cells were incubated for 2 weeks with different concentrations of CI. Colonies were fixed with 4% paraformaldehyde, following with staining by crystal violet. The number of colonies was counted in indicated time periods.

#### 2.1.5 Cell cycle analysis

Cell Cycle Detection Kit (KeyGEN Biotech, China) was used to analyze the cell cycle distribution in accordance with manufacturer’s instructions. U2OS and MG63 cells were seeded at a density of 2 × 10^5^ cells per well in 6-well plates, cultured overnight, and then treated with CI for 12 h. Cells were collected, fixed in cold 75% ethanol overnight at −20°C, digested with RNase at 37°C for 30 min, stained by PI at 37°C for 30 min away from light, then detected on BD FACSCalibur flow cytometer (BD Biosciences, New Jersey, United States).

#### 2.1.6 Apoptosis assay

Flow cytometry was used to assess cell apoptosis using Annexin V-FITC/PI apoptosis detection kit (BD Biosciences, United States). U2OS and MG63 cells (2 × 10^5^ cells/mL) were seeded into 6-well plates (triplicate in each group), cultured overnight, and then treated with CI for 24 h. The cells were harvested, washed with cold PBS twice, and resuspended in 100 µL of binding buffer. The supernatant cells were incubated with 5 µL Annexin V-FITC and 5 µL PI for 15 min in the dark at room temperature. Binding buffer was added to each sample with 400 μL, then detected by flow cytometry.

#### 2.1.7 RNA-seq analysis

U2OS and MG63 cells with or without CI treatment, then total RNA was extracted with TRIzol reagent. RNA-Seq library construction and sequencing were conducted by Tianjin Novogene Bioinformatic Technology Co., Ltd. (Tianjin, China). We obtained the union of all differentially expressed RNA (Including microRNA, mRNA, lncRNA and other RNA information). Illumina Hiseq platform in Novogene Genomics was used for high throughput sequencing. The parameters of |logFC| > 1 and *p*-value <0.05 were used as the screening criterion for differential genes. PPI interactions of the co-downregulated genes were analyzed by Cytoscape software (the larger the node, the higher the degree). The red nodes represented the core position of the PPI network.

#### 2.1.8 PPIase isomerase inhibition assay

Chymotrypsin-coupled PPIase assay was used to determine inhibition of PIN1 isomerase activity, using PIN1 recombinant protein and suc-AAFP-pNA. In simple terms, 0.5 μg PIN1 recombinant protein was pre-incubated with the indicated concentrations of CI in buffer containing 133 mM Tris-HCI, pH 8.0, 5.5 nM chymotrypsin for 20 min at 4°C. The peptide substrate (Suc-AAFP-pNA peptide substrate, final concentration 50 mM) was added immediately before the assay began. The optical density (OD) was measured using a microplate reader spectrophotometer at 450 nm.

#### 2.1.9 Immunohistochemistry assay

Pathological sections of six patients with OS were provided by Tianjin Hospital. Tissue chip (including pathological tissue of 70 patients with OS) was purchased from Zhongke Guanghua Intelligent Biotechnology Co., Ltd. (Cat. L714901). In full compliance with national legislation and ethics (IRB00001052-08044), all patients signed informed consent forms for sample collection and analysis.

The pathological sections were deparaffinized, rehydrated, followed high-pressure antigen retrieval with 10 mM citrate buffer, then blockage of endogenous peroxidase activity by 3% hydrogen peroxide. After washing the sections with PBS and blocking them with BSA, they were incubated with the antibody PIN1, YAP, TAZ and Ki67 at 4°C overnight. Then, they were washed in PBS and incubated with secondary antibodies at room temperature for 1 h. Immunoreactivity was detected using a DAB Kit (Boster Bio, China). The sections were counterstained, dehydrated, sealed, and observed under ECLIPSE Ci-L microscope (Nikon, Tokyo, Japan).

#### 2.1.10 Western blot assay

Standard western blot assays were conducted as described previously (Kong et al., 2022). The antibodies for PIN1 (Cat. No: Ab191271), YAP (Cat. No: Ab56701), TAZ (Cat. No: Ab224239), PI3K (Cat. No: 4292S), AKT (Cat. No: 9272S), p-AKT (Cat. No: 9271S), BAX (Cat. No: 2772S), BCL-2 (Cat. No: 4223S), cleaved-caspase 3 (Cat. No: 9661T), Caspase 3 (Cat. No: 9662), CDK4 (Cat. No: D963E), CDK1 (Cat. No: 77055S), cyclinD1 (Cat. No: 2922S) and cyclinB1 (Cat. No: 4138S) were obtained from Cell Signaling Technology (Boston, United States). The antibody for β-actin was from Beyotime Biotechnology (Shanghai, China). The antibody for GAPDH (Cat. No: 60004-1-Ig) were obtained from Proteintech (Rosemount, IL, United States).

#### 2.1.11 GFP/pcmv-ha-pin1 transfection

Cells were seeded into 6-well culture plates (2 × 10^5^ cells/well) for 24 h, cells were transfected with GFP/pcmv-ha-pin1 for 6 h. Then the cells 24 h after transfection were analyzed under fluorescence microscope (Nikon, Tokyo, Japan) for subsequent experiments.

#### 2.1.12 Molecular docking

The structure of PIN1 (PDB code: 6DUN) was obtained from the protein data bank (http://www.rcsb.org/pdb). We searched for the 2D structure of the 15 potential candidate compounds in CI. Discovery Studio 2019 was used to perform docking simulations of PIN1 and 15 potential candidate compounds for the CDOCKER Experiment. The interaction energies were calculated to predict docking positions, then select the binding pose with the lowest binding energy (kcal mol^−1^).

#### 2.1.13 Statistical analysis

In all experiments, triplicates were performed. Statistical analyses were carried out using Prism software. (GraphPad Prism 6, United States). All the data conformed to the normal distribution and results are expressed as mean ± SEM of at least three independent experiments. Statistical comparisons were performed by Dunnett’s test using one-way ANOVA, two-way repeated-measures ANOVA, non-linear Regression or *t*-test. PIN1 expression correlation with YAP and TAZ was analyzed using the Spearman rank correlation test and an analysis of the data was conducted using SPSS 25.0 software. *p*-value <0.05 was statistically significant.

### 2.2 Results

#### 2.2.1 CI inhibits tumor activity in the OS xenograft mouse model

**FIGURE 1:**
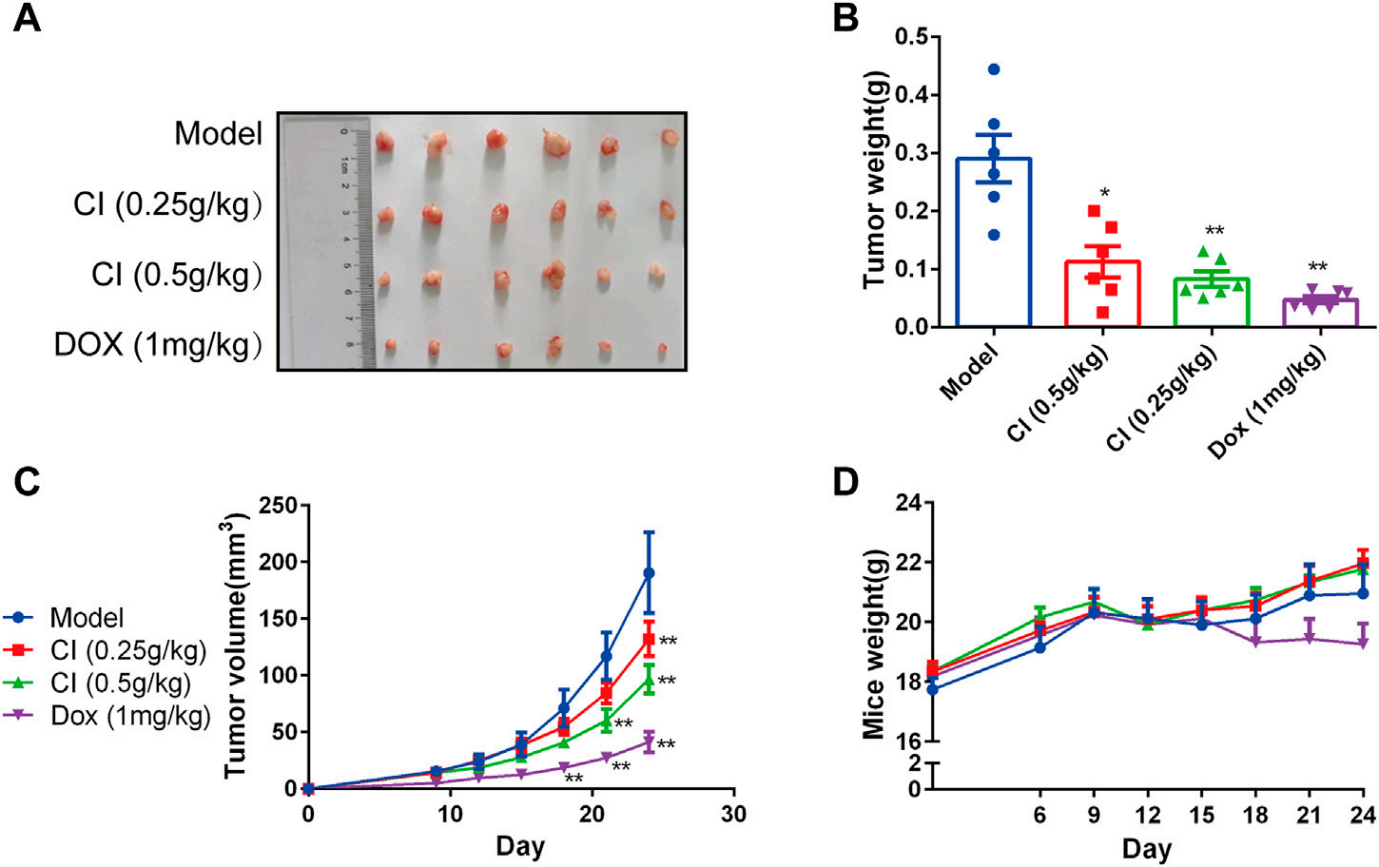
CI inhibits growth and development in ectopic subcutaneous tumor model of U2OS. **(A)** Tumor tissues after sacrifice. **(B)** Tumor weight after CI treatment or DOX. Mean ± SEM. *n* = 6. *p < 0.05, **p < 0.01 vs*.* model group (one-way ANOVA). **(C)** Tumor volume growth curve after CI treatment or DOX. Mean ± SEM. *n* = 6. *p < 0.05, **p < 0.01 vs*.* model group. (two-way repeated-measures ANOVA). **(D)** Mice weight after CI treatment or DOX. Mean ± SEM. *n* = 6. *p > 0.05, vs*.* model group (two-way repeated-measures ANOVA).

BALB/c-nude mice xenografts were established to observe the anti-tumor activity of CI *in vivo*. The treatment group was administrated CI (low dose: 0.25 g/kg; high dose: 0.5 g/kg) or DOX (1 mg/kg) by intraperitoneal injection once a day for a total of 24 days. As shown in Figures 1A–C, CI treatment significantly inhibited tumor growth in terms of both tumor volume and tumor weight, compared with model group. And CI had no significant effect on the body weight of mice (Figure 1D).

#### 2.2.2 CI inhibits OS cells proliferation

**FIGURE 2:**
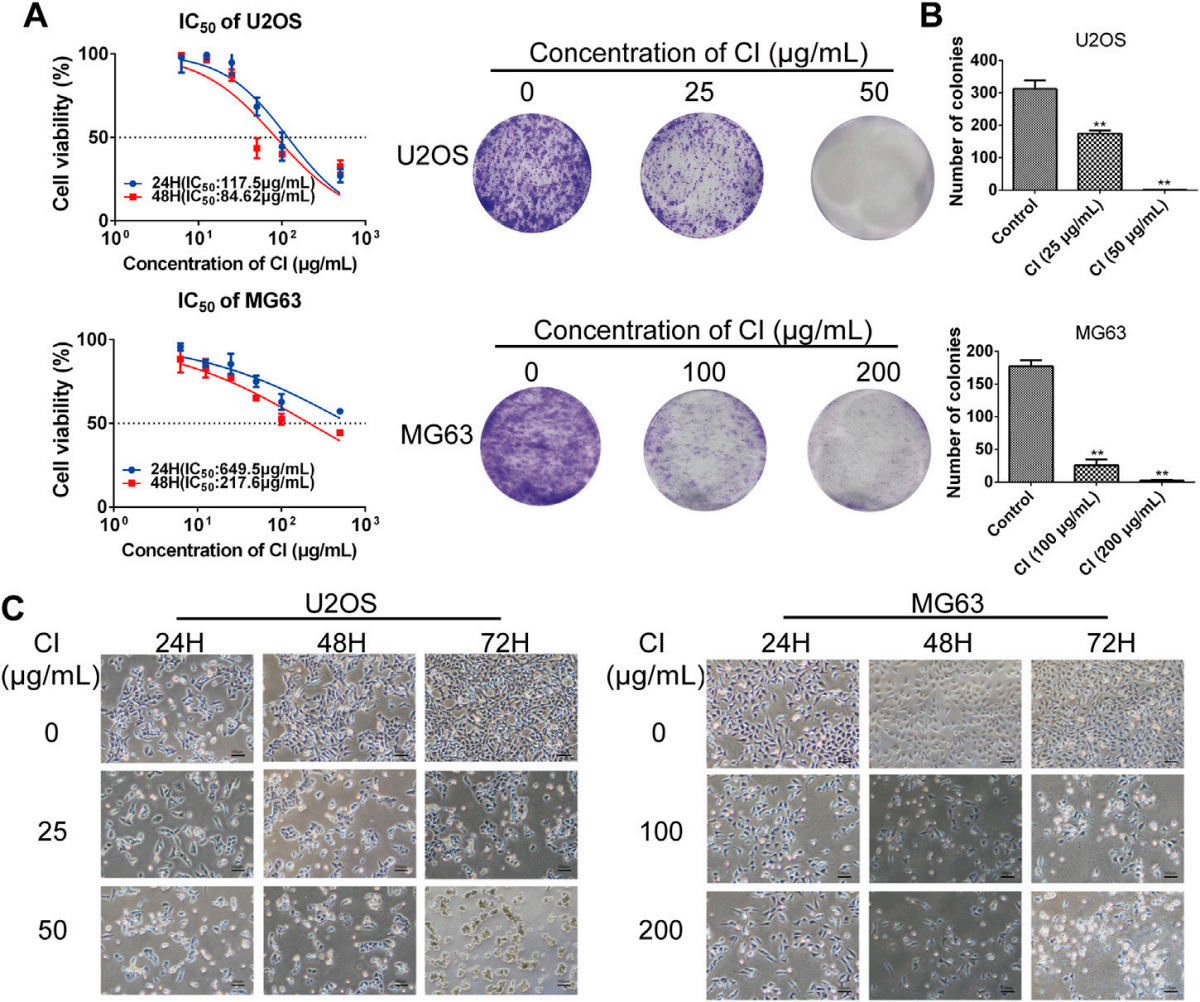
CI reduces cell viability of OS cells. **(A)** After 24, 48 h treatment with CI, the IC_50_ is 117.5 μg/mL, 84.62 μg/mL in U2OS cells and 649.5 μg/mL, 217.6 μg/mL in MG63 cells. Results were presented as the mean ± SEM of three independent experiments. (Non-linear Regression) **(B)** Colony formation assay of U2OS and MG63 cells exposed to CI (0, 25, 50 or 0, 100, 200 μg/mL) for 14 days. The colony numbers (>50 cells/colony) were calculated manually. Results were presented as the mean ± SEM of three independent experiments. *p < 0.05, **p < 0.01 vs*.* control group (one-way ANOVA). **(C)** Morphological changes of U2OS and MG63 cells were treated with CI (0, 25, 50 or 0, 100, 200 μg/mL) for 12, 24 and 48 h.

CCK-8 assay, colony formation, and morphological changes were measured in U2OS and MG63 cells treated with CI. As shown in Figure 2A, CCK-8 assay indicated both OS cells showed significant reductions in viability after exposure to with different concentrations CI of 24 and 48 h, which was dose- and time-dependent. After 48 h treatment with CI, the IC50 is 84.62 μg/mL in U2OS cells and 217.6 μg/mL in MG63 cells. In subsequent experiments, U2OS cells were treated with 25 μg/mL and 50 μg/mL of CI, and MG63 cells were treated with 100 μg/mL and 200 μg/mL of CI. As shown in Figure 2B, the clonogenicity was also decreased after treatment with CI. Apoptotic morphological changes were observed in OS cells following CI exposure, including cell shrinkage, rounding, and granulation. Morphological changes had higher severity in dose- and time-dependent (Figure 2C). We employed the BPH1 (Human Benign Prostatic Hyperplasia) cells and HEK293 (immortalized Human Embryonic Kidney) cells to demonstrate the selective toxicity of CI (Supplementary Figure S2A).

#### 2.2.3 CI induced OS cells cycle arrest and apoptosis

CI is composed of more than 100 monomer compounds (Yu CY et al., 2021), in which bufalin (Jiang et al., 2014; Soumoy et al., 2022; Yu et al., 2022), bufotalin (Zhu et al., 2014; Zhang et al., 2022) and cinobufagin (Pan et al., 2019; Pan et al., 2020), mainly contribute to its anti-cancer activity. Not only CI itself but also its components repress tumorigenesis by regulation of cell proliferation, regulation of apoptotic process, negative regulation of mitotic cell cycle, and G2/M or G1/S transition of mitotic cell cycle (Wang et al., 2020; Li et al., 2022). Dysregulation of cell cycle distribution is an important feature of tumor development, and the induction of apoptosis is often accompanied by cell cycle arrest (Evan and Vousden, 2001). Therefore, we further studied the effects of CI on cell cycle arrest and apoptosis in OS cells.

**FIGURE 3:**
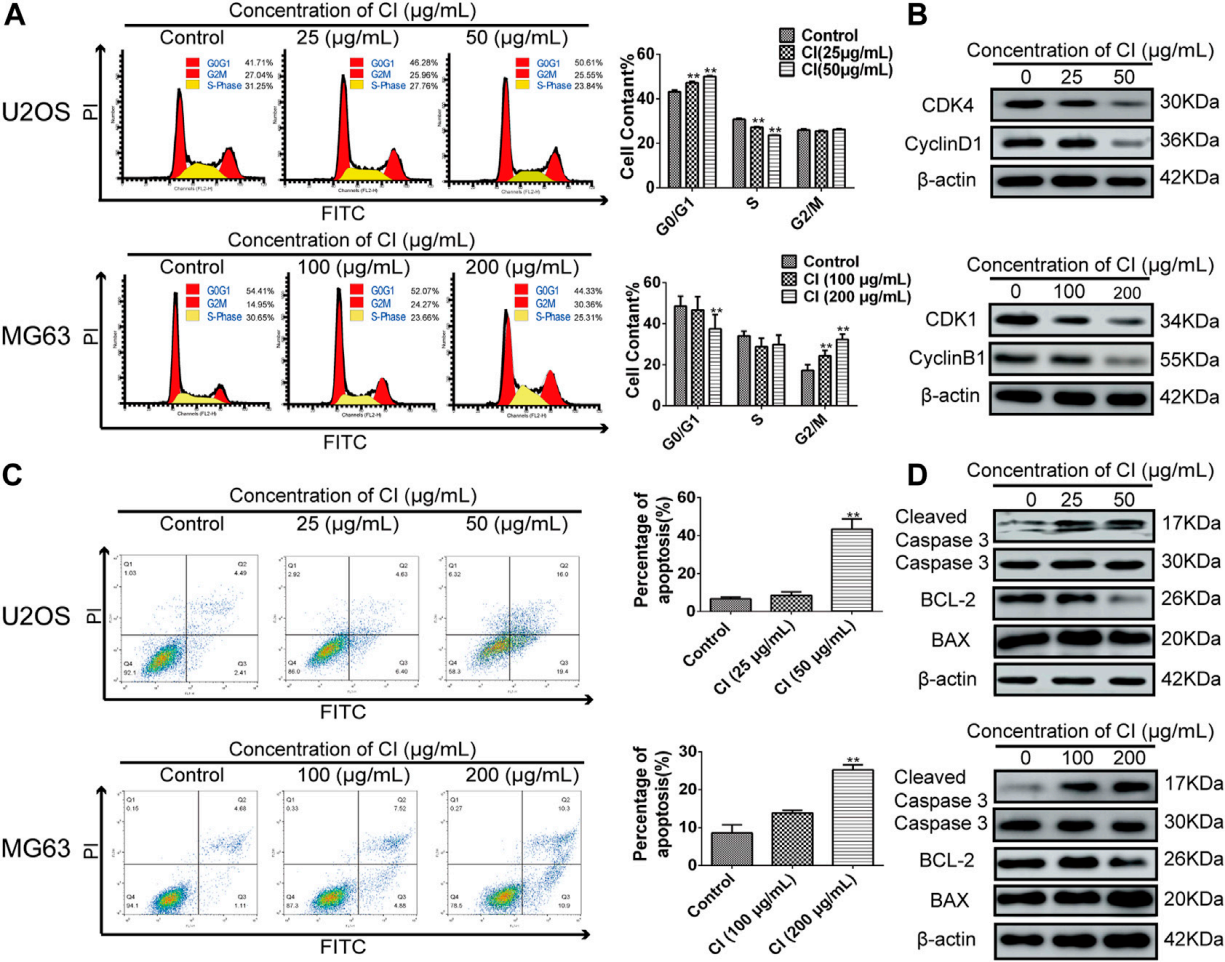
CI induced OS cells cycle arrest and apoptosis. **(A)** Cell cycle phases of U2OS and MG63 cells exposed to CI (0, 25, 50 or 0, 100, 200 μg/mL) for 12 h analyzed using flow cytometry. Results were presented as the mean ± SEM of three independent experiments. *p < 0.05, **p < 0.01 vs*.* control group (one-way ANOVA). **(B)** Levels of expression of CDK4, cyclinD1, CDK1, cyclin B1 were determined using western blot assessment. **(C)** The stained U2OS and MG63 cells exposed to CI (0, 25, 50 or 0, 100, 200 μg/mL) for 24 h. Results were presented as the mean ± SEM of three independent experiments. *p < 0.05, **p < 0.01 vs*.* control group (one-way ANOVA). **(D)** The expression and statistic results of cleaved caspases 3, Caspases 3, BCL-2, and BAX in U2OS and MG63 after CI treatment.

We investigated the effects of CI on cell cycle progression to confirm the relation between growth inhibition and cell cycle arrest. Compared with untreated cells, at given CI after 12 h, a downward trend in S peak and G0/G1 accumulation were observed in U2OS cells. On the other hand, MG63 cells showed a decrease in G0/G1 peak and G2/M accumulation (Figure 3A). Furthermore, western blot results showed that CI treatment clearly downregulated CDK4 and CyclinD1 in U2OS and CDK1 and CyclinB1 in MG63 (Figure 3B). To sum up, by inhibiting the regulation of proteins involved in cell cycle regulation, cell cycle arrest occurred in U2OS at G0/G1 phase and in MG63 at G2/M phase after CI treatment.

According to the morphological changes and cell arrest detected by flow cytometry, apoptosis may participate in the CI treatment of OS. After 24 h treatment with 0 (control), 25 μg/mL and 50 μg/mL of CI in U2OS cells, and with 0 (control), 100 μg/mL and 200 μg/mL of CI in MG63 cells, according to PI and annexin V staining assays, CI induced cell apoptosis in a dose-dependent manner (Figure 3C). In addition, western blot analysis of apoptotic pathway-related proteins showed that CI increased significant cleaved-caspase 3 expression and reduced BCL-2 to BAX ratios (Figure 3D). Overall, in both OS cells, CI induced apoptosis *via* the BCL-2, BAX, and caspase-dependent pathways.

#### 2.2.4 RNA-seq analysis

**FIGURE 4:**
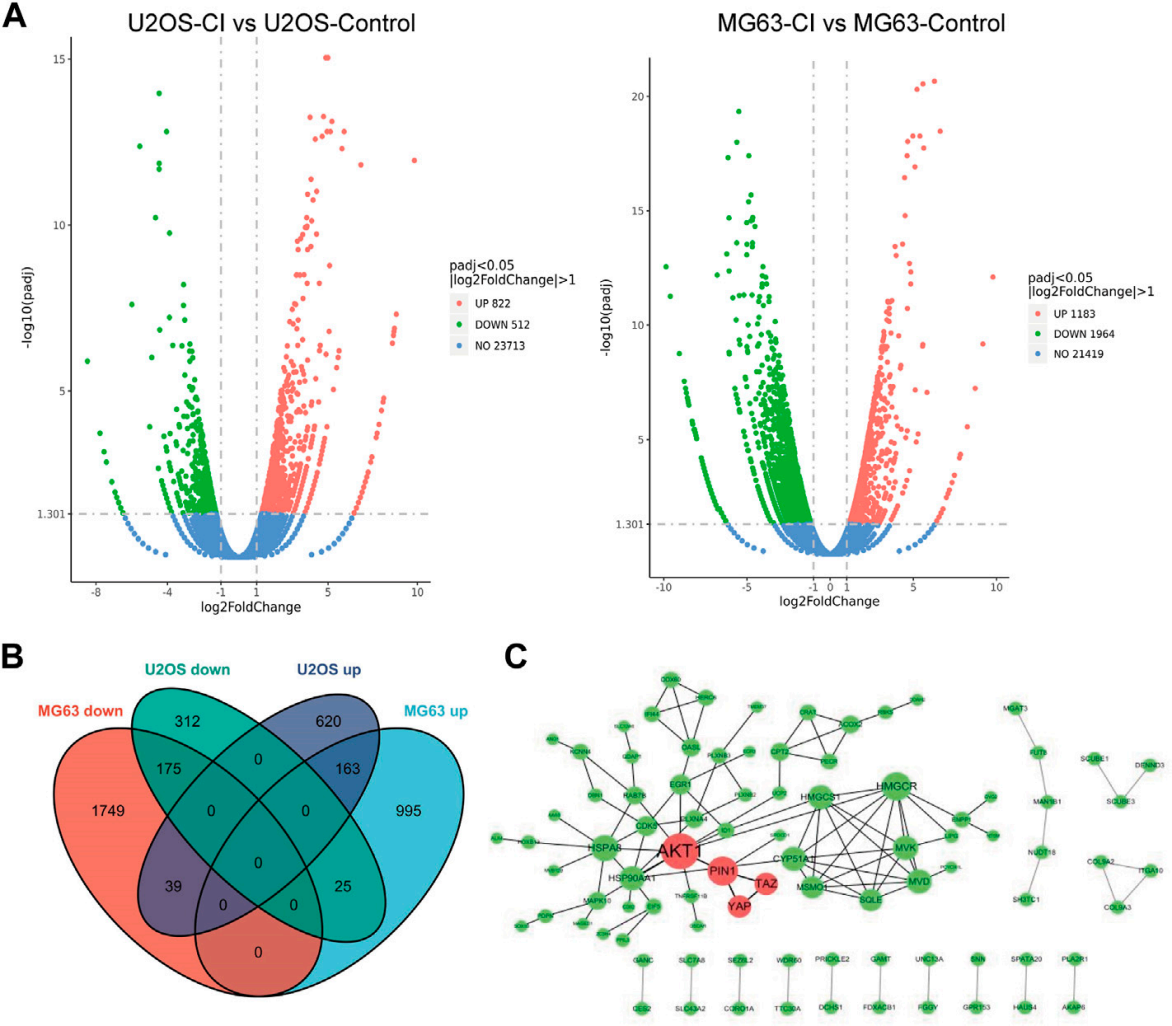
Analysis of CI-regulated gene expression in OS cells. **(A)** Volcano plot of the log2 Fold Change CI/control vs. the -log 10 *p*-value (y-axis) of the genes in U2OS and MG63 cells. **(B)** The intersection of differentially expressed genes between U2OS and MG63 cells were recognized as common differentially expressed genes. **(C)** PPI interactions of the co-downregulated genes were analyzed by Cytoscape software (the larger the node, the higher the degree). The red nodes represented the core position of the PPI network.

An RNA-seq analysis was performed in OS cells to assess CI cellular target engagement. CI treatment regulated 402 genes in both OS cells after 24 h compared to the control. (Figures 4A, B). PPI interactions of the co-downregulated genes were analyzed by Cytoscape software (the larger the node, the higher the degree). The red nodes represented the core position of the PPI network. As can be seen from the results (Figure 4C), Akt, Pin1, Yap, and Taz genes were significantly downregulated. Either PIN1 or YAP/TAZ plays an essential role solely in OS, but PIN1-YAP/TAZ pathway has been poorly addressed so far. In this case, we need to not only verify the expression and correlation of PIN1, YAP and TAZ in OS patients and cells, but also figure out whether CI inhibit PIN1-YAP/TAZ pathway *in vitro* and *in vivo*.

#### 2.2.5 Expression and correlation of PIN1, YAP and TAZ in OS patients and cells

**FIGURE 5:**
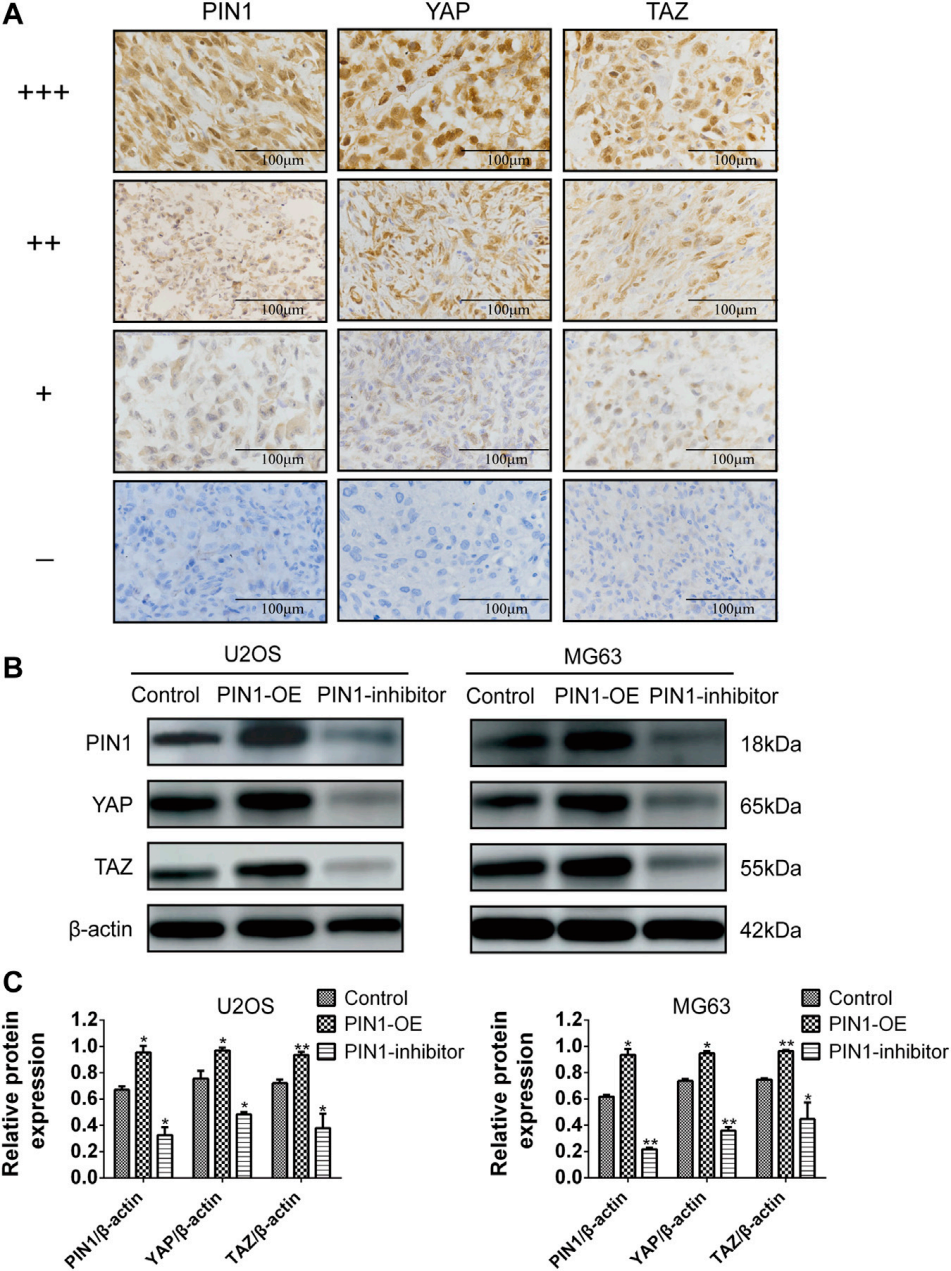
The correlation of PIN1, YAP and TAZ. **(A)** Immunohistochemical results scoring criteria. **(B)** The expression levels of PIN1, YAP, and TAZ were detected by western bolt after PIN1-OE (pcmv-HA-PIN1) and PIN1 inhibitor (juglone). Scale bar stands for 100 μm. **(C)** Quantification of western blot data from **(B)**. Results were presented as the mean ± SEM of three independent experiments. *p < 0.05, **p < 0.01 vs*.* control group (one-way ANOVA).

YAP and TAZ are downstream effect factors of Hippo pathway and regulate the expression of target genes (Cao et al., 2008). Therefore, we mainly put emphasis on the relationship between PIN1 and YAP/TAZ. Firstly, we studied the expression and correlation of PIN1, YAP and TAZ in patients with OS. The expression of PIN1, YAP and TAZ were obtained by positive scoring of pathological sections of 76 OS tissues. We scored the immunohistochemical results (Figure 5A). Spearman rank correlation analysis was conducted on the correlation between PIN1 and YAP, and between PIN1 and TAZ in OS tissues according to the positive histochemical score. PIN1 was positively correlated with YAP expression (r = 0.613, *p* < 0.001) (Table 1). The expression of PIN1 was positively correlated with TAZ (*r* = 0.641, *p* < 0.001) (Table 2). The positive expression rates of PIN1, YAP and TAZ were higher in OS tissues, and PIN1 was positively correlated with the expression of YAP/TAZ.

**TABLE 1:** The correlation of PIN1-YAP was analyzed after IHC results were scored.

PIN1	YAP			
	**−**	**+**	**++**	**+++**
**−**	13	4	1	0
**+**	2	6	6	1
**++**	1	7	18	4
**+++**	1	2	5	5

**TABLE 2:** The correlation of PIN1-TAZ was analyzed after IHC results were scored.

PIN1	TAZ			
	**−**	**+**	**++**	**+++**
**−**	16	2	0	0
**+**	5	6	4	0
**++**	2	13	14	1
**+++**	2	1	9	1

Our experiment investigated the relationship between PIN1 and YAP and TAZ in OS cells by forcing or inhibiting PIN1 expression. Compared with control group, when PIN1 was overexpressed, the expressions of YAP/TAZ were upregulated. On the other hand, PIN1 inhibitor (Juglone) induced the downregulation of YAP/TAZ, which suggesting that PIN1 can act as a positive regulator of key molecules of YAP/TAZ in OS cells (Figures 5B, C).

#### 2.2.6 CI inhibits the PIN1-YAP/TAZ pathway *in vivo* and *in vitro*

**FIGURE 6:**
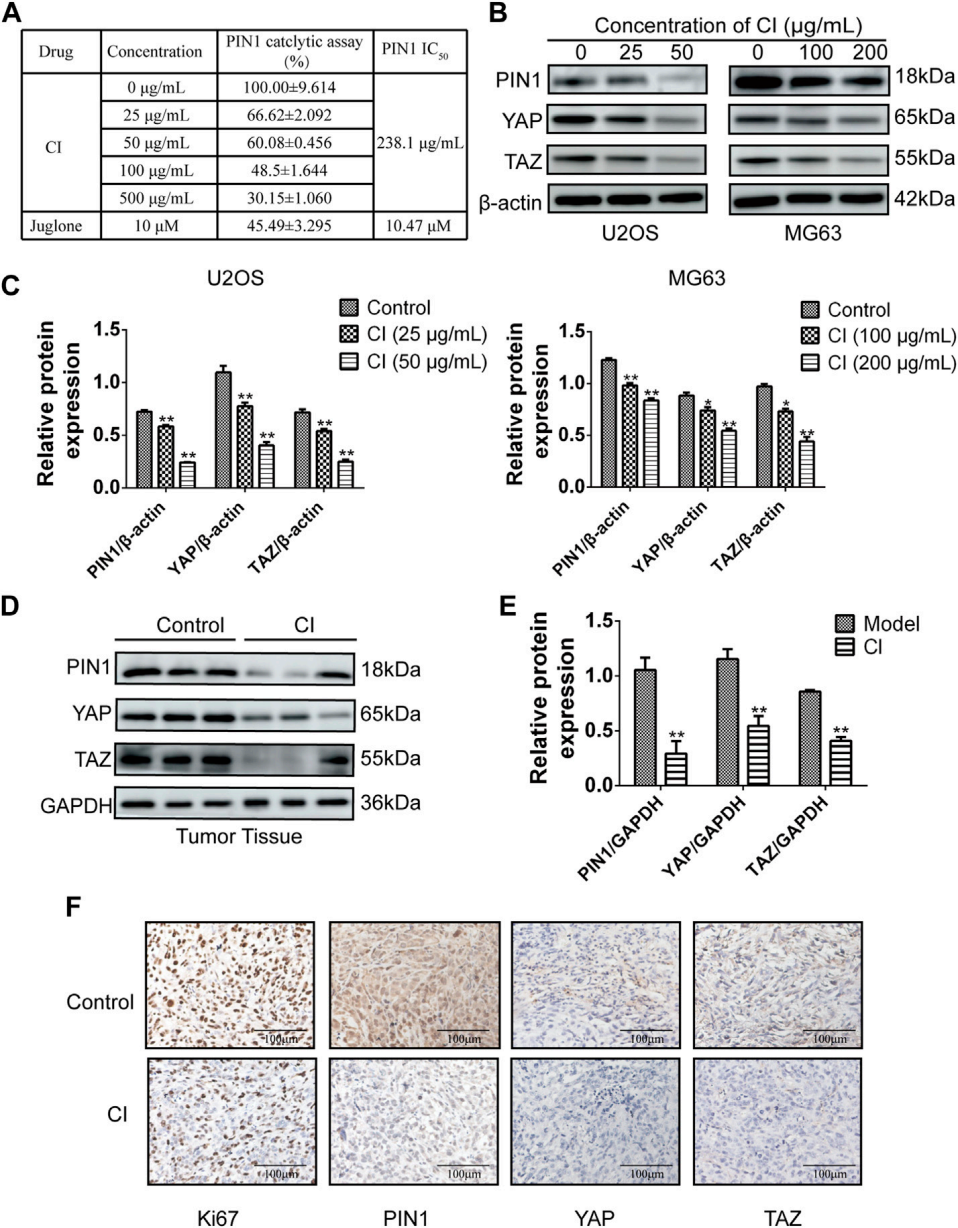
CI inhibits the PIN1-YAP/TAZ pathway *in vivo* and *vitro*. **(A)** PPIase isomerase inhibition assay of PIN1 with CI. Results were presented as the mean ± SEM of three independent experiments. **(B)** The expression levels of PIN1, YAP and TAZ were detected by western bolt after CI treatment in U2OS and MG63 cells. **(C)** Quantification of western blot data from **(B)**. Results were presented as the mean ± SEM of three independent experiments. *p < 0.05, **p < 0.01 vs*.* control group (one-way ANOVA). **(D)** The expression levels of PIN1, YAP and TAZ were detected by western bolt after CI in ectopic subcutaneous tumor model of U2OS. **(E)** Quantification of western blot data from **(D)**. Results were presented as the mean ± SEM of three independent experiments. *p < 0.05, **p < 0.01 vs*.* model group (*t*-test). **(F)** Immunohistochemical analysis of paraffin-embedded U2OS mice tumor tissue sections using antibody (Ki67, PIN1, YAP, TAZ). Scale bar stands for 100 μm.

The PPIase PIN1 controls the isomerization of the Ser/Thr-Pro (pSer/Thr-Pro) motif. We employed PPIase isomerase inhibition assay to evaluate the effect of CI on the binding between PIN1 with its substrate. CI inhibited PIN1 enzyme activity in a dose-dependent manner and it IC_50_ is 238.1 μg/mL (Figure 6A). Next, we further examined the effect of CI on the PIN1-YAP/TAZ pathway *in vitro* and *in vivo*. Compared to the blank control group, both western blot (Figures 6B–E) and immunohistochemistry assays (Figure 6F) showed that the expression of PIN1, YAP and TAZ proteins could be dose-dependently reduced by CI treatment. In conclusion, CI inhibited PIN1 enzyme activity in a dose-dependent manner, followed by impairing the expression of PIN1, YAP and TAZ, thereby exerting anticancer activity. Therefore, our findings suggest that CI can significantly inhibit the proliferation of human OS cells, induce cell cycle arrest and apoptosis, and the mechanism is partly mediated by down-regulating the PIN1-YAP/TAZ signaling pathway. In addition, our study also demonstrated the effect of CI on AKT protein, the expression of PI3K and p-AKT proteins could be dose-dependently reduced by CI treatment *in vitro* (Supplementary Figure S2).

#### 2.2.7 Effects potential candidate compounds of CI on PIN1

**FIGURE 7:**
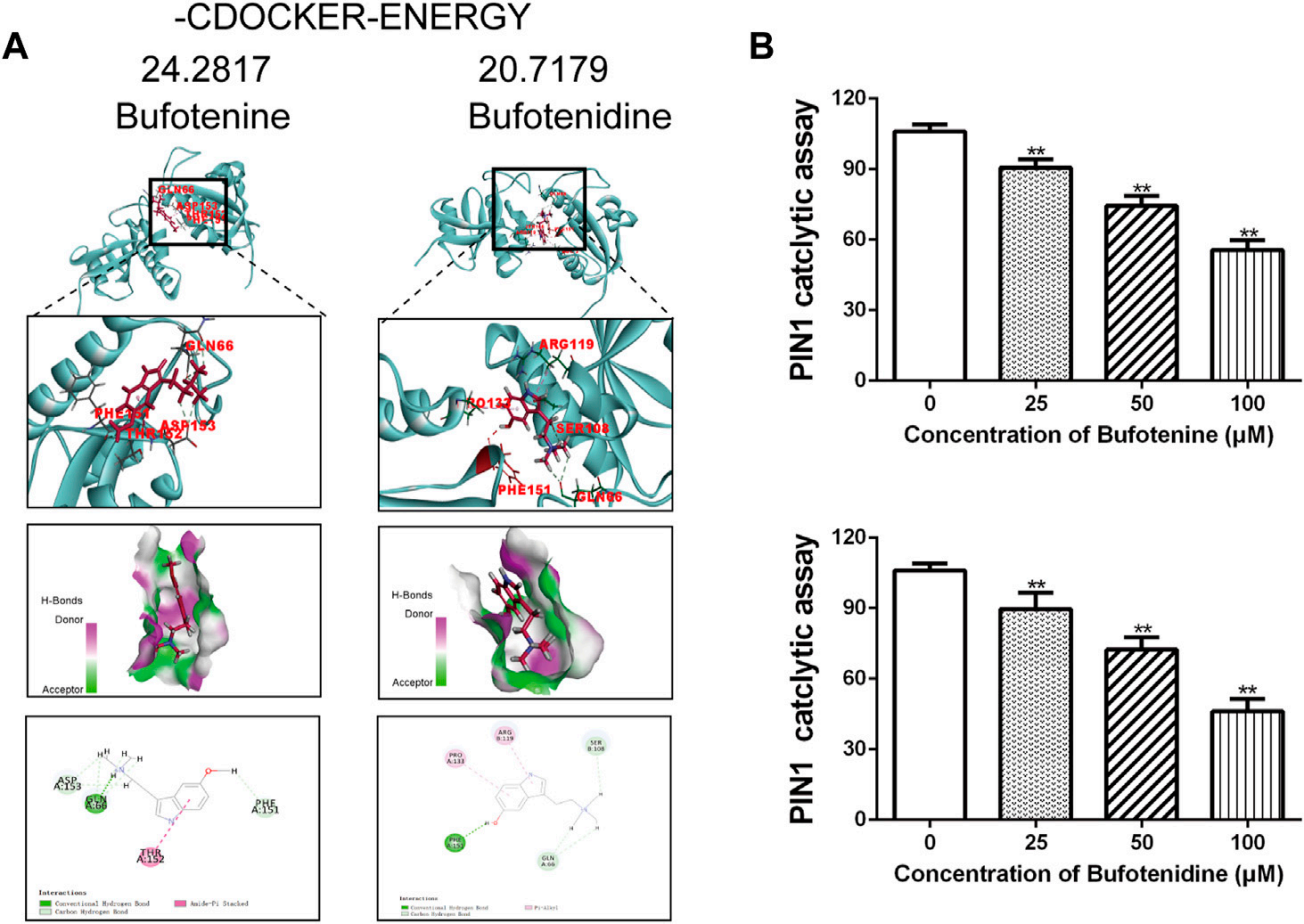
Effects of potential candidate compounds of CI on PIN1. **(A)** Docking simulations of PIN1 with bufotenine and bufotenidine. **(B)** PPIase isomerase inhibition assay of PIN1 with bufotenine and bufotenidine. Data is the average of independent experiments ±SEM. *p < 0.05, **p < 0.01 vs*.* control group (one-way ANOVA).

The function of PIN1 is to bind and modify the three-dimensional conformation of unique phospho-proteins, leading to changes in phosphorylation status and regulation of the structure and folding of proteins. By reviewing the literature, we screened out 15 potential anti-OS candidate components in CI (Yu CY et al., 2021). Meanwhile, CI compounds docking to PIN1 WW and/or PPIase domains, were visualized by Discovery Studio 2019. The CDOCKER docking result showed that all 15 potential candidate compounds could be docked with PIN1 (PDB code: 6DUN). Among them, the two compounds with the lowest docking energy required for PIN docking were bufotenine and bufotenidine (Supplementary Table S1). They occupied the PIN1 kinase domain to inhibit the activity function of PIN1 (Figure 7A). We employed PPIase isomerase inhibition assay to evaluate the effect of bufotenine and bufotenidine on the binding between PIN1 with its substrate. The results show that they inhibited PIN1 enzyme activity in a dose-dependent manner (Figure 7B). These results suggest that these compounds contribute to CI inhibition on PIN1 enzyme activity.

## 3 Discussion

According to traditional Chinese medicine, OS is caused by Yang-Qi deficiency, stagnation of Qi, blood stasis, and cancer toxin stasis (Si and Ding, 2015). Cutis Bufonis is used to treat tumors, sores, and carbuncles due to its ability to clear heat and remove toxicity, induce diuresis, and relieve flatulence. CI, the extract of Cutis Bufonis, is widely used to treat advanced tumors in clinical trials (Qiu and Dong, 2010; Ni et al., 2019). CI has been found to inhibit OS proliferation in modern pharmacological studies (Yin et al., 2013), which was also verified in our study. At the same time, we further verified its anticancer activity *in vivo* by constructing ectopic subcutaneous OS model.

BCL2 and BCL2L1 are the transmembrane molecules in the mitochondria and belongs to the BCL-2 family, which are critical for the intrinsic mitochondrial apoptotic pathway (Kaufmann et al., 2016). Dysregulation of cell cycle distribution is an important feature of tumor development, and the induction of apoptosis is often accompanied by cell cycle arrest. CI induced cell cycle arrest in U2OS at G0/G1 phase but in MG63 at G2/M phase. *P53* gene is wild types in U2OS, but aberrant in MG63. Notably, *p53* plays such a key role in cell cycle controlling (Cappadone et al., 2015; Ho et al., 2021; Hu et al., 2021), that might directly influence different steps of the cell cycle. On the other hand, compounds induce the steps of cell cycle arrest in a dose-dependent manner (Chaiputtanapun et al., 2022). The different concentration of CI in both cell lines may also contribute to its effects on cycle arrest.

Multiple signaling pathways are involved in cancer cell proliferation and downregulation of apoptosis, including PIN1-Hippo pathway, but its role in OS progression remains unclear. The current understanding of PIN1-YAP/TAZ pathway comes from studies performed in breast cancer (Khanal et al., 2019), we firstly identified their relationship in OS. In this study, immunohistochemistry was performed on pathological sections of six patients with OS and tissue chips containing pathological tissues of 70 patients with OS. The expression of PIN1, YAP and TAZ were obtained by positive scoring of pathological sections of 76 OS tissues. The positive signal of PIN1 was brownish yellow and expressed in the nucleus, and the positive expression rate in OS tissues was 76.32%. The positive signal of YAP was brown and expressed in the nucleus or cytoplasm, and the positive expression rate in OS tissues was 77.63%. TAZ positive signal was brownish yellow and expressed in the nucleus or cytoplasm, with a positive expression rate of 67.11% in OS tissues. The results showed that PIN1, YAP, and TAZ were highly expressed in OS tissues, and PIN1 was positively correlated with the expression of YAP and TAZ. We further analyzed the expression of PIN1, YAP, and TAZ with the age, sex, place of onset, clinical staging, lymph node metastasis and other clinical pathological parameters of patients with OS, and the results were not significantly correlated due to the small number of samples we had. In future, we will further enrich the sample size and conduct further research.

PIN1 is the only phosphorylation-dependent peptidyl-prolyl cis-trans isomerase known in the human proteome (Lu and Zhou, 2007). A PIN1-mediated isomerization affects substrate stability, subcellular localization, activity, and binding to interaction partners, including proline-directed kinases and phosphatases (Dubiella et al., 2021). PIN1 is overexpressed and/or overactivated in tumors, with poor clinical prognosis (Tan et al., 2010). Through inhibition of PIN1, it is possible to block multiple cancer-driving pathways with limited side effects (Zhou and Lu, 2016). PIN1 inhibitors, such as juglone, arsenic trioxide (ATO), all-trans retinoic acid (ATRA) and KPT-6566, exhibit anticancer activity. However, their lack of specificity and/or cell permeability makes them unreliable tools for evaluating the pharmacological inhibition of PIN1 *in vivo* (Hennig et al., 1998; Wei et al., 2015; Campaner et al., 2017; Kozono et al., 2018). The underlying molecular pathogenesis are distinguished between breast cancer and OS patients, which leads to uncommon clinical treatment. Intriguingly, CI is clinically used in both diseases, we verified that PIN1 is one of the key molecules, which almost acts as the center to regulate the multiple cancer-driving processes. Our findings demonstrated that CI was not only anti-OS *in vivo* and *in vitro*, but also inhibited PIN1 expression.

PIN1 inhibitors, including KPT-6566 and juglone, play their roles not only by inhibition the PPIase isomerase activity of PIN1 *via* covalently interacting with its catalytic core, but also by decreasing PIN1 at protein level *via* inducing the degradation of PIN1 (Hennig et al., 1998; Campaner et al., 2017). In this study, PPIase isomerase inhibition assay indicated that CI inhibited the activity of PPIase isomerase in a dose-dependent manner. And further Western blot assay confirmed the expression of PIN1 proteins could be dose-dependently reduced by CI treatment at protein level. Interestingly, RNA-seq data showed that PIN1 was significantly downregulated, which suggested that CI could downregulate PIN1 at the transcriptional level. Traditional Chinese medicine is characterized by multiple-components and multiple-targets, so PIN1 may be regulated at different molecular levels and protein levels through multiple components to multiple targets and multiple pathways. We are conducting in-depth research on the monomer components of CI, which may provide us more clues in future.

Molecular docking was used to assess the potential impact of 15 compounds of CI on PIN1 activity. We ranked the interaction energies required for each monomer to bind PIN1 (Supplementary Table S1). To predict docking positions, interaction energies were calculated, then the binding pose with the lowest energy was selected. As a rule, the lower the interaction energy, the better the binding ability. Bufotenine and bufotenidine, the top two in rank, the docking energy were 24.2817 kcal mol^−1^ and 20.7179 kcal mol^−1^. The docking energy required for PIN docking of juglone is 21.522 kcal mol^−1^. The results of PPIase isomerase inhibition assay showed that bufotenine and bufotenidine inhibited PIN1 enzyme activity in a dose-dependent manner. These results suggest that 15 compounds may be involved in the pharmacological effect of CI on inhibiting PIN1 against OS to varying degrees.

## 4 Conclusion

**FIGURE 8:**
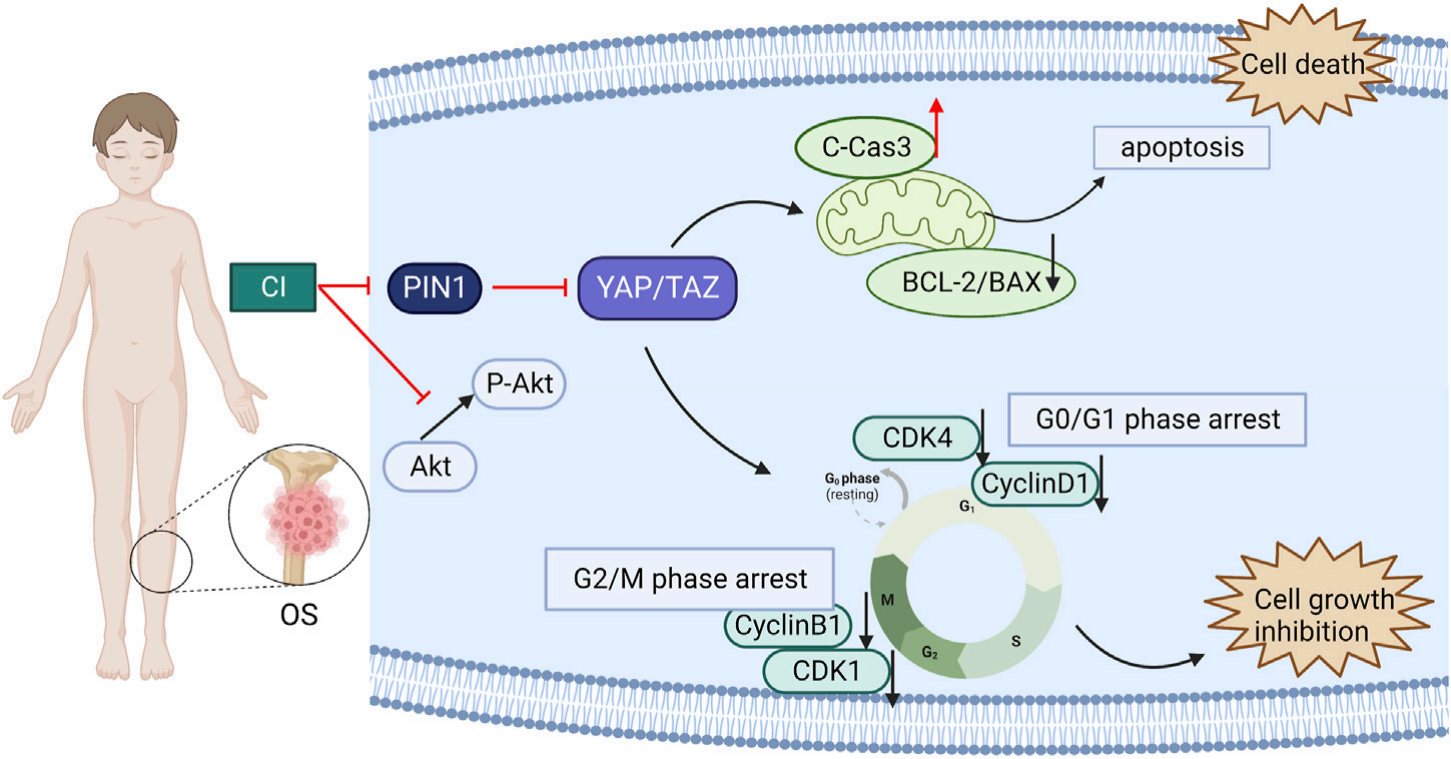
A schematic diagram of the effect of CI on human OS cells. CI induced G2/M or G0/G1 cell cycle arrest and apoptosis in human OS cells through inhibition the PIN1-YAP/TAZ signaling pathway.

In our experiments, we first confirmed that CI inhibited tumor growth in U2OS xenograft mice, and reduced cell proliferation of OS cells, U2OS and MG63, in a dose-and time-dependent manner. And CI induced cell cycle arrest and apoptosis in human OS cells. Then, the correlation of PIN1-YAP/TAZ in OS was found and proved by RNA-seq and immunohistochemistry of clinicopathological tissue. Finally, our findings suggest that CI can exert its anti-OS effect through the PIN1-YAP/TAZ pathway. Moreover, both bufotenine and bufotenidine are the valuable PIN1 inhibitors for the treatment of OS (Figure 8).

## Data Availability

The datasets presented in this study can be found in online repositories. The names of the repository/repositories and accession number(s) can be found in the article/Supplementary Material.

## Supplementary material

SUPPLEMENTARY TABLE S1 Interaction energies ranking of 15 compounds with PIN1 molecule docking.

SUPPLEMENTARY FIGURE S1 BPH-1 and HEK293 cells were treated with CI (5, 50, 500 µg/mL) for 24, 48 h and cell viability was determined by CCK8 assay. Results were presented as the mean ± SEM of three independent experiments. **p* < 0.05, ***p* < 0.01 vs. control group (one-way ANOVA).

SUPPLEMENTARY FIGURE S2 The expression levels of PI3K, p-AKT and AKT were detected by western bolt.

SUPPLEMENTARY FIGURE S3 Docking simulations of PIN1 and 13 potential candidate compounds and juglone.
