# Supplementary material for: Cinobufacini injection suppresses the proliferation of human osteosarcoma cells by inhibiting PIN1-YAP/TAZ signaling pathway
Source: Front Pharmacol. 2023 Mar 17;14:1081363. doi: 10.3389/fphar.2023.1081363 (PMC10063998; doi:10.3389/fphar.2023.1081363)
Supplement: Supplementary file 1 [file Table1.docx]

**Supplementary Table**

**Supplementary Table 1 Interaction energies ranking of 15 compounds with PIN1 molecule docking**

| Compound | -CDOCKER-ENERGY  (kcal mol^−1^) |
| --- | --- |
| Bufotenine | 24.2817 |
| Bufotenidine | 20.7179 |
| Bufothionine | 12.593 |
| Dehydrobufotenine | 9.62918 |
| Gamatin | 1.97725 |
| Cinobufagin | -26.6954 |
| Resibufogenin | -27.521 |
| Bufotalinin | -35.2778 |
| Bufotalin | -37.1937 |
| 5β-Hydroxybufotalin | -42.1286 |
| Hellebrigenin | -45.5489 |
| Arenobufagin | -46.8793 |
| Bufalin | -47.8257 |
| Cinobufotalin | -50.5781 |
| Hellebrigenol | -56.9561 |
