# Supplementary figures and images for: Cinobufacini injection suppresses the proliferation of human osteosarcoma cells by inhibiting PIN1-YAP/TAZ signaling pathway
Source: Front Pharmacol. 2023 Mar 17;14:1081363. doi: 10.3389/fphar.2023.1081363 (PMC10063998; doi:10.3389/fphar.2023.1081363)

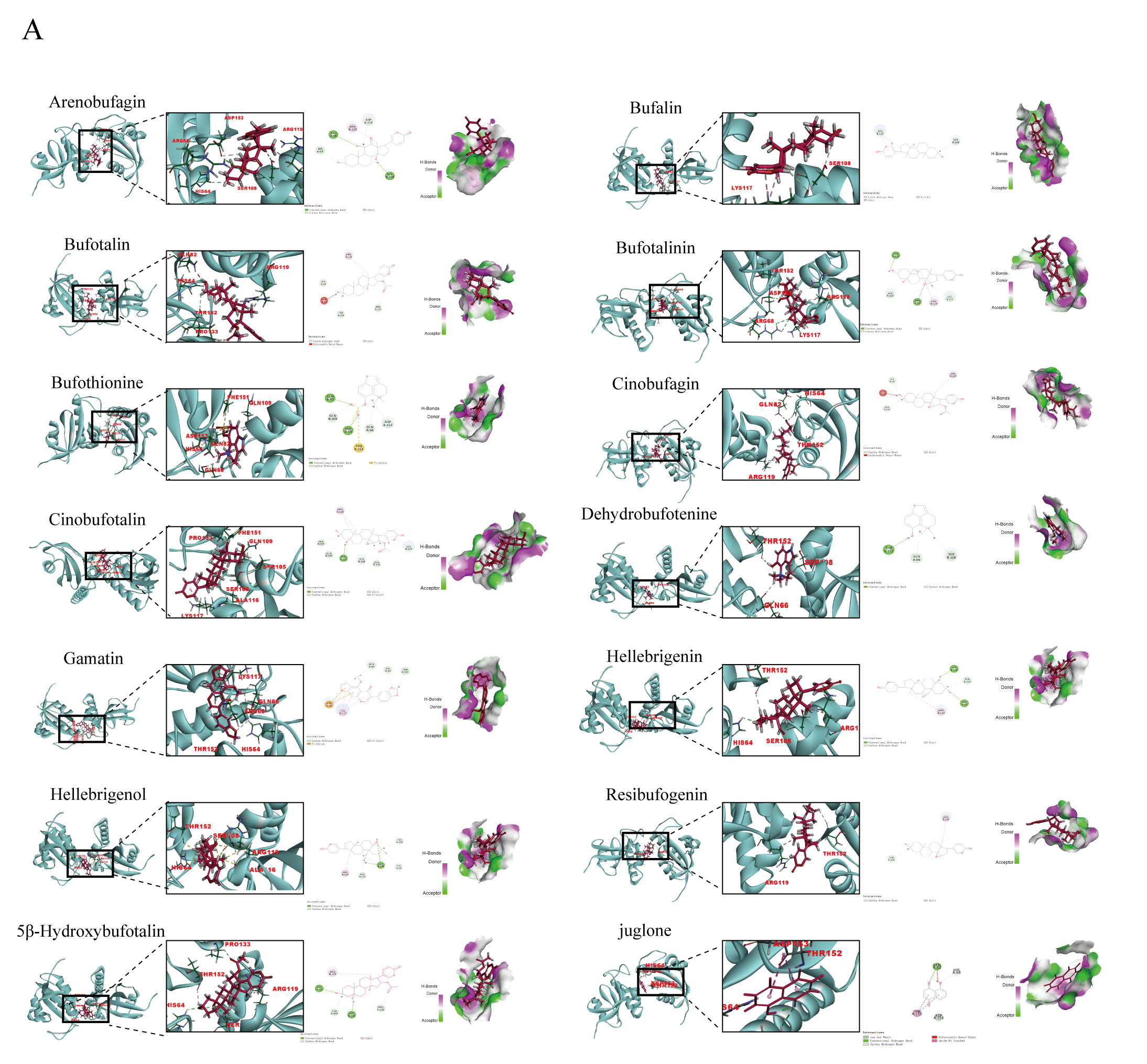

Supplement: Supplementary file 2 [file Image3.TIF]

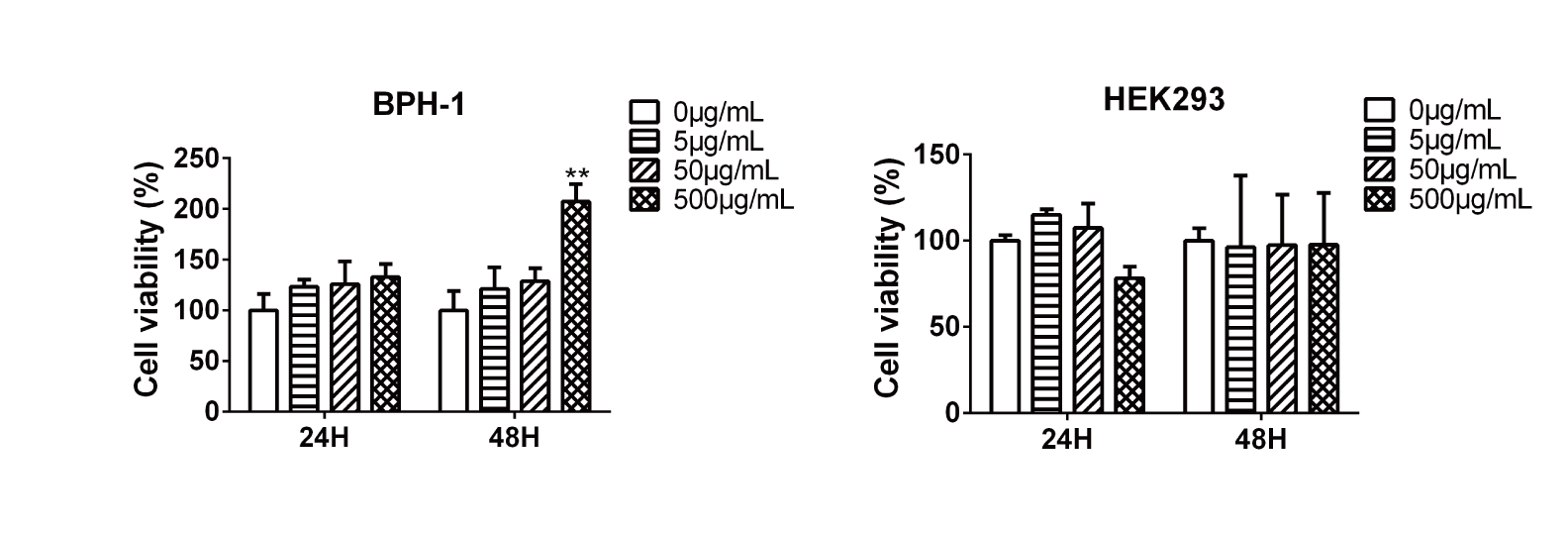

Supplement: Supplementary file 3 [file Image2.TIF]

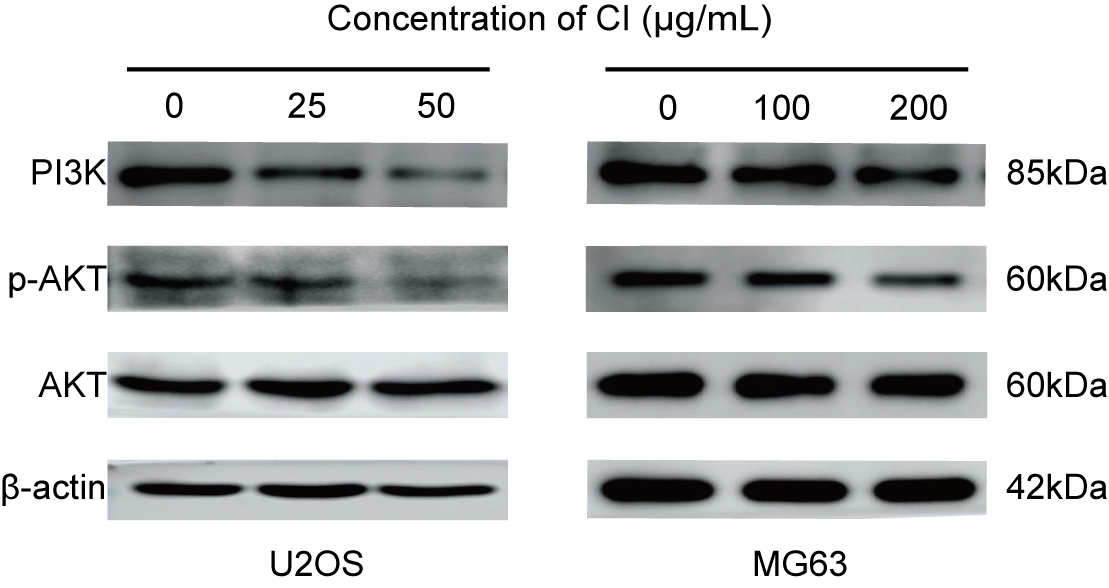

Supplement: Supplementary file 4 [file Image1.TIF]
